# The impact of water management and nitrification inhibitors on methane emissions from paddy soil

**DOI:** 10.1371/journal.pone.0346521

**Published:** 2026-04-10

**Authors:** Wenli Cui, Xiaoxiao Li, Wenjun Jin, Ning Zhou, Zhaorong Dong

**Affiliations:** College of Agriculture, Anhui Agricultural University, Hefei, Anhui; Center for Research and Technology Transfer, VIET NAM

## Abstract

Methane (CH₄) is a potent greenhouse gas, and rice paddies are a major source of agricultural CH₄ emissions. Water management and nitrification inhibitors can significantly affect CH_4_ emissions, but their coupled effects on rhizospheric CH₄ emissions remain unclear. This study employed a rhizobox experiment to investigate the effects of three water management practices—continuous flooding irrigation (C), wetting irrigation (W), and alternate wetting and drying irrigation (A)—and the nitrification inhibitor DMPP (3,4-dimethylpyrazole phosphate) on CH₄ emissions from rice paddies. The results revealed that the rhizosphere was the primary zone for CH₄ emissions, with fluxes up to eight times greater than those from the bulk soil. In all the treatments, peak CH₄ emissions consistently occurred during the rice maturity stage. This peak is attributed to increased carbon availability from root senescence, coupled with soil redox conditions primarily dictated by the respective water management regime. Water management emerged as the primary driver of emission reduction. Compared with C, A significantly reduced cumulative CH₄ emissions by approximately 83.0% in the bulk soil and by 96.9% in the rhizosphere. The application of DMPP further enhanced this mitigation effect, particularly under W and C conditions, leading to an additional 23.06% to 35.94% reduction in bulk soil CH₄ emissions. The observed mechanisms of DMPP addition likely extend beyond its traditional role as a nitrification inhibitor. In conclusion, alternating wetting and drying irrigation with DMPP addition most effectively suppressed CH₄ emissions by synergistically regulating water–carbon–nitrogen coupling processes. This study highlights the central role of rhizospheric processes in reducing emissions, providing a deeper theoretical foundation for developing targeted low-carbon management strategies for rice paddy cultivation.

## Introduction

Water resources represent one of Earth’s most invaluable natural assets and are fundamental to both human sustenance and agricultural productivity. In China, where agriculture forms the bedrock of national development, the role of water in ensuring food security is paramount. Globally, agricultural water consumption accounts for nearly 70% of freshwater resources; this figure is approximately 80% of the total water withdrawal in China. As a leading rice producer, China contributes approximately 35% of global rice output, yet this comes at the cost of consuming an estimated 70% of the world’s agricultural irrigation water. However, increasing global population growth and the pervasive impacts of climate change are intensifying water scarcity, presenting a formidable obstacle to sustainable agricultural development worldwide. Consequently, the imperative for water conservation has become critically important for future human development [1–3].

Under conditions of increasing water scarcity, alternate wetting and drying (A) irrigation and wetting irrigation (W) have emerged as crucial water-saving strategies in rice production. Numerous studies have consistently demonstrated that continuous flooding (C) during the rice growth period creates highly anaerobic conditions in paddy fields, which in turn significantly promotes methane (CH₄) production and emission [4]. Conversely, water-saving irrigation practices, such as A and W, increase soil permeability. This allows for greater diffusion of atmospheric oxygen (O₂) into the soil, disrupting the prevailing reducing environment and consequently inhibiting CH₄ production. Thus, compared with C, water-saving irrigation can substantially reduce CH₄ emissions throughout the rice growth period. For instance, compared with continuous flooding, Sass et al. [5] reported that intermittent irrigation substantially reduced CH₄ emissions from rice paddies. Cai et al. [6] reported that continuous flooding during rice growth resulted in much higher CH₄ emissions than in fields subjected to mid-season drainage or alternate wetting and drying. Yagi et al. [7] studied the effects of continuous flooding and water drainage on CH₄ emissions and reported that several short-term drainage periods during rice growth significantly reduced CH₄ emissions. Yu Feng et al. demonstrated that compared with conventional irrigation, alternate wetting and drying irrigation throughout the growth period significantly reduced CH₄ emissions, and the combination of alternate wetting and drying with nitrification inhibitors further decreased CH₄ emissions [8–9]. These findings collectively underscore that transitioning from traditional continuous flooding to water-saving irrigation practices is an effective strategy for mitigating CH₄ emissions from rice paddies.

DMPP is recognized as a novel and efficient nitrification inhibitor, primarily known for its ability to significantly reduce nitrate (NO₃ ⁻ -N) leaching. The inhibitory effect of DMPP on nitrification is intricately linked to various soil properties, including pH, organic carbon content, and total nitrogen content. In addition to its impact on nitrogen dynamics, DMPP application significantly impacts greenhouse gas emissions. Numerous studies have indicated that the application of DMPP in field experiments or incubation experiments can reduce CH₄ emissions by 25%–55%. Related research [10] suggests that DMPP improves nitrogen use efficiency by reducing nitrogen loss and promotes rice root development and that well-developed roots can increase radial oxygen loss, increase soil redox potential, and inhibit methanogen activity, thereby reducing CH₄ emissions. Schimel et al. [11] found that when the NH₄ ⁺ concentration is high in rice paddies, the utilization of CH₄ by methanotrophs has a dual effect: On the one hand, a high NH₄ ⁺ concentration may inhibit methanotroph activity, leading to increased CH₄ emissions; on the other hand, NH₄ ⁺ might also stimulate the growth and activity of methanotrophs, thereby reducing CH₄ emissions. Zhang Xianxian et al. [12] revealed that DMPP inhibits the conversion of NH₄ ⁺ -N to NO₃ ⁻ -N, i.e., nitrification. During this process, the utilization of CH₄ by methanotrophs is relatively lower than that of NH₄ ⁺ , thus improving nitrogen fertilizer use efficiency and significantly reducing CH₄ emissions in rice paddies.

CH₄ emissions from rice paddies are influenced by a complex interplay of environmental and management factors. The irrigation method directly affects CH₄ emissions; water-saving irrigation can significantly reduce CH₄ emissions. However, water-saving irrigation also intensifies the nitrification process of soil nitrogen, thereby affecting nitrogen fertilizer use efficiency. Applying nitrification inhibitors can regulate soil nitrogen transformation, achieving the goal of improving nitrogen use efficiency while saving water. However, the application of nitrification inhibitors affects soil nitrogen concentration and crop growth, which might in turn influence CH₄ emissions. Currently, the specific mechanisms and influencing factors of nitrification inhibitors on CH₄ emissions under different water-saving irrigation conditions require further in-depth research. In this experiment, three water management practices—continuous flooding irrigation, wetting irrigation, and alternate wetting and drying irrigation—were compared to clarify their regulatory effects on overall and rhizospheric methane emissions in rice paddies, the spatiotemporal distribution characteristics of methane emissions under different irrigation conditions were analyzed, and the inhibitory effect of DMPP application on methane emissions was investigated. The study focuses on elucidating the mechanism by which DMPP affects methanogen substrate supply and community structure by regulating rhizospheric dissolved organic carbon (DOC) and key nutrient availability. We hypothesize that a synergistic effect exists between alternate wetting and drying irrigation and DMPP application. This synergy primarily regulates the methanogen community structure and promotes methane oxidation rather than simply inhibiting methane production [13–17].

## Materials and methods

### Site description and soil

The experiments were conducted at the Nongcuiyuan Experimental Base of Anhui Agricultural University (31°87′N, 117°25′E). The soil was collected from a rice–wheat rotation field at the Guohe Experimental Station of Anhui Agricultural University (31°48′N, 117°23′E) (soil type: sandy loam; Gleyic Stagnic Anthrosol). Soil was taken from the 0–20 cm layer at wheat maturity, passed through a 4-mm sieve, and mixed homogeneously for use.

### Experimental design

Zhendao 18 (Japonica rice, Oryza sativa L. subsp. japonica) was used as the test crop and was raised in a dry seedbed nursery. Uniform seedlings were selected and transplanted manually, with three seedlings per rhizobox. The sowing date was May 12, 2021; the transplanting date was June 23, 2021; and the rice grains were harvested on October 6, 2021. Water management is described in Section 1.2. On the basis of local soil conditions, fertilizers were applied at rates of 300 N kg hm^-2^, 135 kg P_2_O_5_ kg hm^-2^, and 270 kg K_2_O kg hm^-2^. All the phosphorus and potassium fertilizers were applied as the base fertilizer and mixed into the soil. Nitrogen fertilizer (urea) was applied in three splits: base fertilizer:tillering fertilizer:panicle fertilizer = 4:3:3. The base fertilizer was applied on June 23, the tillering fertilizer on July 7, and the panicle fertilizer on August 15. Pest and disease control was applied as needed on the basis of field conditions.

Rhizoboxes made of PVC pipes were used. Each rhizobox consisted of two compartments: an outer bulk soil region and a central rhizosphere soil region. The bulk and rhizosphere soils were separated by a 30-μm nylon mesh, which effectively prevented root penetration while allowing the exchange of water and nutrients.

On the basis of previous survey data of soil bulk density (0–20 cm layer) from paddy fields near the soil collection site, the soil bulk density in the rhizoboxes was set at 1.11 g cm^-3^ (n = 14). According to this bulk density, the mass of soil equivalent to the 0–20 cm layer was calculated and packed into the rhizoboxes and compacted to the target bulk density. The rhizoboxes were buried 20 cm deep in the soil to prevent drastic internal temperature fluctuations. Each treatment involved 40 rhizoboxes. A plastic canopy was set up over the experimental area to prevent rainfall effects.

A two-factor completely randomized design was adopted. Factor 1 was water management, with three treatments: continuous flooding irrigation (C), wetting irrigation (W), and alternate wetting and drying irrigation (A). The soil water level was monitored via perforated pipes installed in the rhizoboxes and replenished promptly. The specific water depth/potential conditions were as follows:

Continuous flooding (C): A ~ 3 cm water layer was maintained, except during the mid-season drainage period and one week before harvest when the soil was drained.

Wetting irrigation (W): Soil moisture was maintained without standing water, except during mid-season drainage and one week before harvest. Specifically, the soil water level was kept between −1 cm and +1 cm (the soil surface was 0 cm). When the water level decreased to −1 cm, + 1 cm of water was added, and the cycle was repeated after natural drainage to −1 cm.

Alternate wetting and drying (A): This treatment was initiated after seedling recovery. The soil water level alternated between −10 cm and +3 cm. When the level decreased to −10 cm, water was added to +3 cm, and the cycle was repeated after natural drainage to −10 cm [18–20].

Factor 2 was the application or non-application of 3,4-dimethylpyrazole phosphate (DMPP). DMPP (purity ≥97%, calculated as 97% for use) was provided by Shanghai Xianding Biotechnology Co., Ltd. The DMPP treatment involved the application of DMPP with base, tillering, and panicle fertilizers; the control treatment received no DMPP. DMPP was applied at 2% of the nitrogen fertilizer amount, which is sufficient for experimental requirements.

### Sampling and measurements

Gas samples were collected using the static closed-chamber method. The CH₄ concentration in the gas samples was determined by gas chromatography (Agilent 7890A; Agilent Technologies, USA) equipped with an FID detector (detector temperature 300 °C; column temperature 60 °C). Chambers and bases were made of PVC. Bases were installed for both the central and outer sections of the rhizobox. Two chamber sizes (internal diameters: 24 cm and 10.5 cm, initial height 50 cm, later increased to 100 cm) were used for sampling bulk and rhizosphere CH₄ emissions, respectively. The internal diameter of the external base was 25 cm, and that of the central base was 11 cm. Chambers were wrapped with sponge and aluminum foil to minimize internal temperature fluctuations. Each chamber was equipped with a separate temperature probe, fan, and sampling port.

Gas sampling commenced after base fertilizer application and was conducted between 8:30 AM and 11:30 AM. Before sampling, the bases were filled with water to ensure an airtight seal. After placing the chamber, 50 ml gas samples were taken at 0, 7, 14, and 21 minutes, and the internal chamber temperature was recorded simultaneously. For each sampling event, three replicate rhizoboxes per treatment were randomly selected. Surface soil temperature and moisture were measured concurrently during gas sampling. A 5TE sensor was inserted into the surface soil, and the data were logged using an EM50 data logger (Decagon, USA). Soil temperature at a depth of 5 cm was continuously monitored using HOBO loggers (MX2201, Onset, USA). Surface soil samples were promptly taken to the laboratory. NH_4_^+^–N and NO_3_^-^–N were extracted with 1 mol L^-1^ KCl solution and determined using a discrete autoanalyzer (CleverChem380, Germany). Approximately 10 g of fresh soil was oven-dried at 105 °C to a constant weight to determine the soil moisture content.

### Statistical analysis

The CH₄ emission flux (F) was calculated using the following formula:


$$\text{F}=\frac{\text{dC}}{\text{dt}}\times \text{H}\times \frac{\text{M}\times \text{P}}{\text{R}\times (\text{273}+\text{T})}$$ (1)

where:

F is the gas emission flux (μg m ⁻ ² h ⁻ ¹; note the unit conversion from the provided μg cm ⁻ ² min ⁻ ¹ for consistency with the results),

dC/dt is the rate of change in the CH₄ concentration mixing ratio during the closure period (μmol mol ⁻ ¹ min ⁻ ¹),

H is the height of the chamber (cm),

M is the molar mass of CH₄ (16 g mol ⁻ ¹),

R is the universal gas constant (8.314472 cm³ kPa K ⁻ ¹ mol ⁻ ¹; using consistent units),

P is the atmospheric pressure at the sampling site (kPa, assumed standard 101.325 kPa if not measured),

T is the average temperature inside the chamber during sampling (°C).

dC/dt was determined from the slope of the linear regression of the CH₄ concentration against time using the four sampling points. Cumulative emissions were calculated by integrating the emission fluxes over time.

The CH₄ emission flux from the bulk soil (FNR) was calculated as follows:


$${\text{F}}_{\text{NR}}=\frac{{\text{F}}_{\text{T}}\times {\text{S}}_{\text{T}}-{\text{F}}_{\text{R}}\times {\text{S}}_{\text{R}}}{{\text{S}}_{\text{NR}}}$$ (2)

where:

FNR is the bulk soil CH₄ emission flux,

FT is the whole plot (bulk + rhizosphere) CH₄ emission flux,

ST is the area of the whole plot,

FR is the rhizosphere soil CH₄ emission flux,

SR is the area of the rhizosphere region,

SNR is the area of the bulk soil region.

Data statistics, analysis, and graphing were performed using EXCEL 2013, R (version 4.1.2), and Origin 2018. Multiple comparisons were analyzed using two-way ANOVA and the least significant difference (LSD) test (p < 0.05).

## Results

### Effects of water management and DMPP on methane emission fluxes

**Fig 1:**
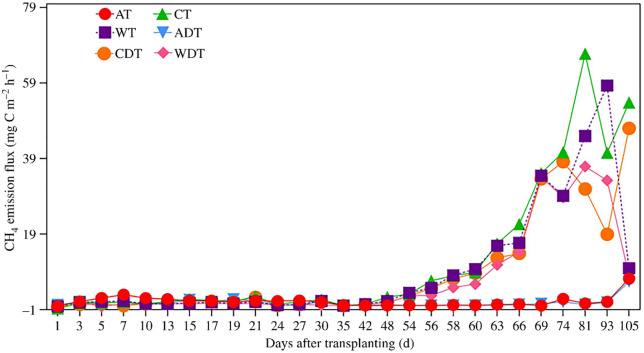
Effects of DMPP on total methane emissions under different water management practices (A: alternate wetting and drying irrigation; B: wetting irrigation; C: continuous flooding irrigation; D: DMPP addition; T: total. The data are presented as the means ± standard errors).

As shown in Fig 1, for the bulk soil CH₄ emission flux under treatment A without DMPP, a distinct peak occurred 7 days after transplanting (DAT) after base fertilizer application, and the highest flux during the entire growth period was observed just before harvest. Under the W treatment without DMPP, the flux increased markedly after panicle fertilizer application, peaking at 93 DAT, and then decreased rapidly during maturity. Under treatment C without DMPP, the peak flux occurred at 81 DAT. With respect to DMPP application, under treatment A, the peak bulk flux occurred before harvest. Under treatment W with DMPP, a distinct peak appeared at 69 DAT, and the highest flux occurred at 81 DAT, followed by a rapid decrease. Under treatment C with DMPP, a distinct peak appeared at 74 DAT, after which it decreased but then increased again, peaking just before harvest. The ranges of bulk soil CH₄ flux with and without DMPP were as follows: treatment A: 0.08–6.41 and −0.08–7.18 mg C m ⁻ ² h ⁻ ¹; treatment W: −0.20–36.91 and −0.16–58.37 mg C m ⁻ ² h ⁻ ¹; and treatment C: −0.23–47.05 and −0.73–66.77 mg C m ⁻ ² h ⁻ ¹, respectively.

**Fig 2:**
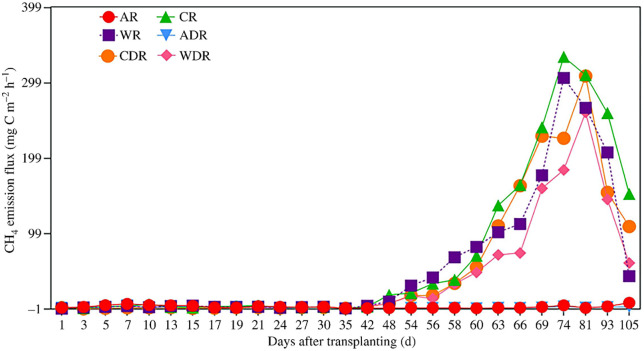
Effects of DMPP on rhizosphere methane emissions under different water management practices (A: alternate wetting and drying irrigation; W: wetting irrigation; C: continuous flooding irrigation; R: rhizosphere; D: DMPP addition. The data are presented as the means ± standard errors).

As shown in Fig 2, the rhizospheric CH₄ emission flux patterns varied among the treatments. Under treatment A without DMPP, distinct rhizospheric flux peaks occurred at 7 DAT (after base fertilizer) and 23 DAT (after tillering fertilizer), with the highest flux occurring before harvest. With respect to DMPP application in treatment A, distinct peaks occurred at 7 DAT (the highest during the growth period) and 23 DAT. The rhizospheric flux ranges with and without DMPP for treatment A were 0.08–4.06 and 0.13–7.31 mg C m ⁻ ² h ⁻ ¹, respectively. Under treatments W and C without DMPP, the trends were similar initially, with low rhizospheric fluxes and a sharp decrease noted during mid-season drainage, followed by a gradual increase after drainage, peaking at 76 DAT for both. With DMPP application under treatments W and C, the rhizospheric fluxes were initially low, increased gradually after mid-season drainage, and peaked at 81 DAT for both treatments. The rhizospheric flux ranges with and without DMPP were as follows: treatment W: 0.02–260.08 and −0.42–305.78 mg C m ⁻ ² h ⁻ ¹; treatment C: −0.67–308.36 and 0.45–333.80 mg C m ⁻ ² h ⁻ ¹, respectively.

### Effects of water management and DMPP on cumulative methane emissions

**Fig 3:**
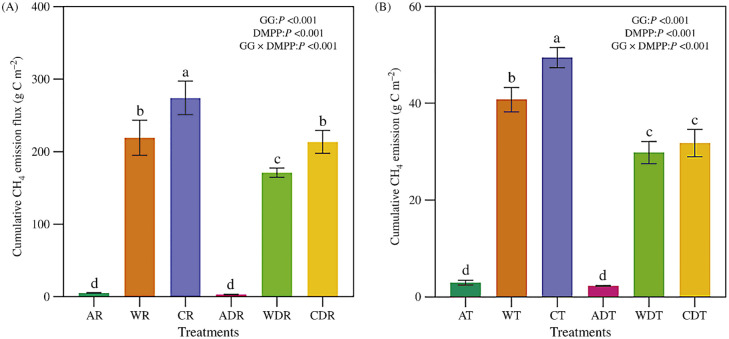
Effects of DMPP on cumulative methane emissions under different water management practices (A: alternate wetting and drying irrigation; B: wetting irrigation; C: continuous flooding irrigation; + DMPP: DMPP addition; R: rhizosphere; T: total. The data are presented as the means ± standard errors. Different lowercase letters represent significant differences between treatments at the 0.05 level.).

As shown in Fig 3, water management and DMPP significantly affected cumulative rhizospheric and bulk soil CH₄ emissions. Compared with treatment A, treatments W and C significantly increased cumulative bulk soil CH₄ emissions. DMPP application significantly reduced cumulative bulk soil emissions in treatments W and C. DMPP reduced cumulative bulk soil CH₄ emissions by 23.06%, 26.80%, and 35.94% in treatments A, W, and C, respectively. The cumulative bulk soil emissions with and without DMPP were A: 2.29 and 2.98 g C m ⁻ ²; W: 29.80 and 40.72 g C m ⁻ ²; and C: 31.66 and 49.41 g C m ⁻ ², respectively.

For cumulative rhizospheric CH₄ emissions, the order was C > W > A. DMPP application significantly reduced cumulative rhizospheric emissions in treatments W and C but had no significant effect in treatment A. The cumulative rhizospheric emissions with and without DMPP were A: 3.06 and 5.15 g C m ⁻ ²; W: 160.70 and 219.13 g C m ⁻ ²; and C: 213.42 and 274.12 g C m ⁻ ², respectively.

As shown in Table 1, within 14 days after base fertilizer application, DMPP significantly reduced cumulative bulk and rhizospheric CH₄ emissions under treatment A; DMPP had no significant effect on treatment W; and DMPP had no significant effect on bulk soil emissions but significantly reduced rhizospheric emissions under treatment C. Within 14 days after tillering fertilizer application, DMPP had no significant effect on bulk soil emissions under any water management but significantly reduced rhizospheric emissions under treatment C. Within 14 days after panicle fertilizer application, DMPP significantly reduced bulk and rhizospheric emissions in treatments W and C but had no significant effect in treatment A. During other periods of the maturity stage, DMPP significantly reduced bulk soil emissions in treatments W and C and significantly reduced rhizospheric emissions in treatment C.

**Table 1:** Effects of DMPP on cumulative CH4 emissions during different periods under different water management practices.

Treatments	During 14 days after application of basal fertilizer		During 14 days after application of tiller fertilizer		During 14 days after application of spike fertilizer		Other period	
	Total cumulative CH_4_ emission (g C m^-2^)	Cumulative CH_4_ emission of rhizosphere (g C m^-2^)	Total cumulative CH_4_ emission (g C m^-2^)	Cumulative CH_4_ emission of rhizosphere (g C m^-2^)	Total cumulative CH_4_ emission (g C m^-2^)	Cumulative CH_4_ emission of rhizosphere (g C m^-2^)	Total cumulative CH_4_ emission (g C m^-2^)	Cumulative CH_4_ emission of rhizosphere (g C m^-2^)
A	0.49 ± 0.27a	1.06 ± 0.52a	0.46 ± 0.20a	0.78 ± 0.27a	0.08 ± 0.07d	0.25 ± 0.15d	1.77 ± 0.40d	2.75 ± 0.27c
W	0.21 ± 0.03b	0.50 ± 0.07bc	0.23 ± 0.02b	0.74 ± 0.22a	3.37 ± 0.27ab	24.97 ± 2.63ab	36.61 ± 4.18b	191.45 ± 38.90b
C	0.15 ± 0.03 b	0.75 ± 0.29ab	0.48 ± 0.04a	0.76 ± 0.28a	3.67 ± 0.30a	26.53 ± 2.07a	44.67 ± 3.79a	244.16 ± 37.58a
AD	0.21 ± 0.01b	0.46 ± 0.24bc	0.36 ± 0.13ab	0.58 ± 0.25ab	0.07 ± 0.04d	0.24 ± 0.26d	1.48 ± 0.11d	1.52 ± 0.64c
WD	0.11 ± 0.08b	0.48 ± 0.04bc	0.18 ± 0.03b	0.57 ± 0.03ab	2.32 ± 0.64c	14.86 ± 4.20c	27.05 ± 4.06 c	143.66 ± 12.53b
CD	0.08 ± 0.03b	0.14 ± 0.02c	0.38 ± 0.05ab	0.22 ± 0.12b	2.82 ± 0.58bc	21.52 ± 1.53b	28.53 ± 4.18c	190.58 ± 27.84b
F–value								
GG	6.716*	2.493	9.098**	1.400	116.373***	186.199***	196.156***	120.424***
DMPP	7.516*	10.141*	3.310	8.894*	11.787**	21.992***	30.710***	6.622*
GG×DMPP	1.419	2.730	0.130	1.299	2.976	7.332**	8.655**	1.677

Note: A: alternate wetting and drying irrigation; W: wetting irrigation; C: continuous flooding irrigation; GG: water management; D: DMPP. ***, **, and * indicate significance at the 0.001, 0.01, and 0.05 levels, respectively. The data are presented as the means ± standard errors. Different lowercase letters indicate significant differences at the 0.05 level among treatments.

## Discussion

### Impact of water management on soil methane emissions

This study revealed significant differences in CH₄ emissions among the three water management modes, in the order of continuous flooding > wetting irrigation > alternate wetting and drying. This aligns well with the previous research consensus [13,21,22]. Alternate wetting and drying irrigation creates periodic aerobic soil conditions, directly inhibiting the activity of anaerobic methanogens but significantly promoting the activity of methanotrophs, thereby curbing CH₄ at both the production and oxidation stages.

A more critical finding is that rhizospheric CH₄ emission fluxes significantly surpassed bulk soil fluxes, especially under wetting and continuous flooding. Under these conditions, the cumulative rhizospheric emissions were 5–8 times greater than those from the bulk soil. This substantial difference highlights the rhizosphere as the predominant zone for CH₄ production. Consequently, treating paddy fields as a homogeneous system for emission assessments could lead to a severe underestimation of the contribution of rhizospheric processes. Rice roots not only continuously supply readily decomposable carbon to methanogens via exudates and sloughed-off cells but also create an efficient conduit for CH₄ transport to the atmosphere through their aerenchyma. Therefore, CH₄ mitigation strategies should primarily target this core rhizosphere region.

The following is an image illustrating the rhizosphere and CH₄ emissions:

In terms of the timing of peak emissions, this study revealed the highest fluxes during the late growth stage (maturity), which is consistent with reports by Li Daoxi [13] and Zhang Xianxian [12]. This is likely due to the aging and death of rice roots and root exudates, which provide abundant carbon sources, promote microbial activity, and are coupled with effective transport pathways, leading to emission peaks. Research by Yang Guangming et al. [23–24] also revealed high CH₄ emission peaks just before rice harvest, primarily because of the release of CH₄ accumulated in the soil. However, further investigation is needed to determine whether the peak is driven solely by an absolute increase in carbon source quantity or by the cessation of radial oxygen loss due to declining root activity, which results in the creation of a stricter anaerobic microenvironment, or both. This suggests that the overall redox potential (Eh), governed by water management, is likely a more fundamental controlling factor than the availability of carbon sources. When the bulk soil is in an oxidized state, the increased carbon from root senescence is buffered by a strong oxidative capacity. Conversely, under the reduced conditions of continuous flooding, any additional carbon input can be efficiently converted to CH₄.

### Interactive effects of water management and DMPP on methane emissions

DMPP, as an efficient nitrification inhibitor, could maintain higher ammonium nitrogen levels in soil, therefore reducing nitrate formation and subsequent denitrification losses and N₂O emissions. However, this study revealed that DMPP significantly inhibited CH₄ emissions (reducing bulk soil emissions by 23.06–35.94% and rhizospheric emissions by 21.95–40.67%), with these effects being more pronounced under the more water-saturated continuous flooding and wetting irrigation treatments. Why would an inhibitor targeting the nitrogen cycle so effectively influence the carbon cycle-centric process of CH₄ production? We propose that this mechanism may involve DMPP indirectly triggering “carbon–nitrogen coupling limitation” in the rhizosphere microzone. By inhibiting nitrification, DMPP maintains higher NH₄ ⁺ concentrations. Some studies have suggested that high NH₄ ⁺ concentrations may have direct toxic effects on certain methanogenic archaeal species [32] or increase CH₄ consumption by stimulating methanotroph activity. DMPP might alter the rhizospheric microbial community structure, for instance, by promoting the growth of heterotrophic nitrifiers or other microbes that utilize organic carbon, thereby competitively consuming carbon sources otherwise available to methanogens. This “carbon source competition” warrants further investigation. Related studies [25–32] have indicated that soil moisture content significantly influences the effectiveness of DMPP. In the alternate wetting and drying treatment, the mitigation effect of DMPP was relatively weaker and nonsignificant during specific post-fertilization periods. This is likely because the strongly oxidizing environment of treatment A itself already greatly suppresses methanogenesis, making the additional effect of DMPP less apparent. Conversely, under continuous flooding and wetting irrigation, DMPP interferes with the rhizosphere through the aforementioned carbon–nitrogen coupling mechanism, resulting in significant mitigation effects. This phenomenon aligns with the view of Pasda et al. [25] that soil moisture affects DMPP adsorption and mobility, thereby influencing its effective concentration. Related research [12,30–33] has reported conflicting findings regarding the effect of DMPP on CH₄ emissions, with some studies indicating reduction and others indicating no effect or even promotion. This study specifically demonstrated that DMPP notably inhibited CH₄ emissions from rice paddies.

## Conclusions

This study provides crucial insights into the pivotal roles of alternating wetting and drying irrigation and nitrification inhibitors in regulating CH_4_ emissions from rice paddies. This research definitively confirms that the rice maturity stage is a critical period for CH₄ emissions, largely driven by the release of dissolved organic carbon (DOC) from senescing roots. Among the three water management practices investigated, A irrigation treatment resulted in the most substantial mitigation potential, leading to significant reductions in both bulk soil and rhizospheric CH₄ emissions. DMPP application further enhanced this mitigation effect, involving reducing the availability of key nutrients and DOC in the rhizosphere, thereby limiting the substrate supply for methanogens. In summary, the combined “alternate wetting and drying irrigation + DMPP” strategy effectively inhibited CH_4_ production by optimizing the water–carbon–nitrogen coupling relationship, providing robust theoretical and technical support for achieving green, low-carbon rice production. However, while the rhizobox method effectively distinguishes rhizospheric and non-rhizospheric processes, its confined microenvironment may differ from actual field conditions. Future research should explore how the coupled interactions of carbon and nitrogen cycles coregulate both CH₄ and nitrous oxide (N₂O) emissions.

## Supporting information

S1 File The dataset underlying the findings presented in this manuscript is provided as a separate file. The data include all relevant experimental measurements, numerical values, and metadata necessary to reproduce the analyses, figures, and conclusions reported in the study. Detailed variable definitions and data structure are described within the file. (XLSX)
